# Biosynthesis of C4–C8 3-Hydroxycarboxylic Acids from Glucose through the Inverted Fatty Acid β-Oxidation by Metabolically Engineered *Escherichia coli*

**DOI:** 10.3390/biom14040449

**Published:** 2024-04-07

**Authors:** Andrey Yu. Gulevich, Alexandra Yu. Skorokhodova, Vladimir G. Debabov

**Affiliations:** Research Center of Biotechnology, Russian Academy of Sciences, 33, bld. 2. Leninsky Ave., Moscow 119071, Russia; sasha.skorokhodova@gmail.com (A.Y.S.); debabov13@yandex.ru (V.G.D.)

**Keywords:** *Escherichia coli*, fatty acid β-oxidation, glucose, 3-hydroxycarboxylic acids

## Abstract

Inverted fatty acid β-oxidation represents a versatile biochemical platform for biosynthesis by the engineered microbial strains of numerous value-added chemicals from convenient and abundant renewable carbon sources, including biomass-derived sugars. Although, in recent years, significant progress has been made in the production through this pathway of n-alcohols, 1,3-diols, and carboxylic acids and its 2,3-unsaturated derivatives, the potential of the pathway for the biosynthesis of 3-hydroxycarboxylic acids remained almost undisclosed. In this study, we demonstrate the microaerobic production of even-chain-length C4–C8 3-hydroxycarboxylic acids from glucose through the inverted fatty acid β-oxidation by engineered *E. coli* strains. The notable accumulation of target compounds was achieved upon the strong constitutive expression of the genes *atoB*, *fadA*, *fadB*, *fadE*/*fabI*, and *tesB*, which code for the key enzymes catalysing reactions of aerobic fatty acid β-oxidation and thioesterase II, in strains devoid of mixed-acid fermentation pathways and lacking nonspecific thioesterase YciA. The best performing recombinants were able to synthesise up to 14.5 mM of 3-hydroxycarboxylic acids from glucose with a total yield of 0.34 mol/mol and a C4/C6/C8 ratio averaging approximately 63/28/9. The results provide a framework for the development of highly efficient strains and processes for the bio-based production of valuable 3-hydroxycarboxylates from renewable raw materials.

## 1. Introduction

**Figure 1:**
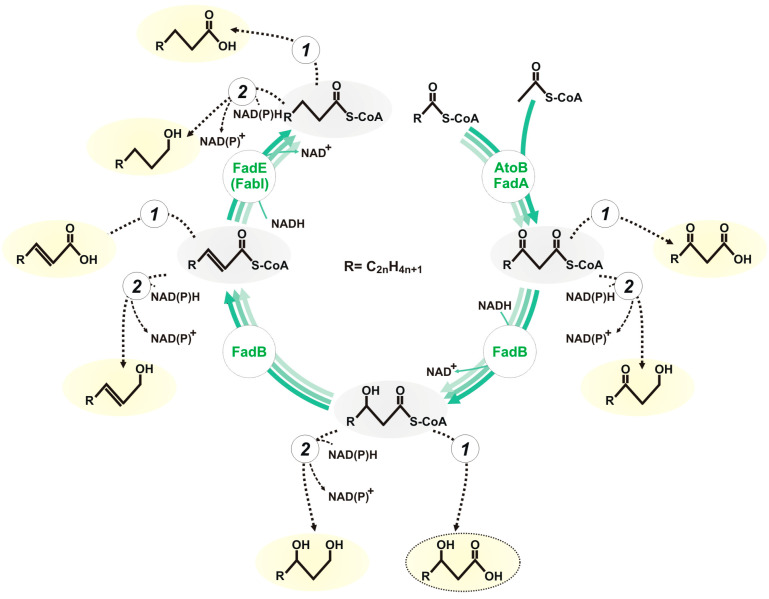
The general scheme of iBOX and its biosynthetic potential for the production of valuable chemicals. The intermediates of the inverted cycle are as follows (listed in clockwise direction): acetyl-CoA/acyl-CoA, 3-ketoacyl-CoA, 3-hydroxyacyl-CoA, enoyl-CoA, and acyl-CoA. Key enzymes in the pathway are indicated by the respective *E. coli* proteins: acetyl-CoA C-acetyltransferases (EC 2.3.1.9 and EC 2.3.1.16), AtoB, FadA; NADH-dependent 3-hydroxyacyl-CoA dehydrogenase (EC 1.1.1.35), FadB; enoyl-CoA hydratase (EC 4.2.1.17), FadB; FADH_2_-dependent acyl-CoA dehydrogenase (EC 1.3.8.-), FadE; NADH-dependent acyl-CoA dehydrogenase. Valuable products are highlighted in yellow. 3-hydroxycarboxylic acids, the target products of the current study, are circled with a dashed oval. Terminating enzymes that generate target compounds from iBOX intermediates are appropriate thioesterases (1) and aldehyde/alcohol dehydrogenases (2).

Fatty acid β-oxidation is the conserved biochemical pathway participating in lipid degradation in a variety of organisms. Upon the utilisation of fatty substrates, this cyclic process proceeds via the sequential conversion of acyl-CoA to 2-enoyl-CoA, the subsequent hydration of 2-enoyl-CoA to 3-hydroxyacyl-CoA, followed by the oxidation of the 3-hydroxy group leading to 3-oxoacyl-CoA formation, and the final thiolysis resulting in the release of an acetyl-CoA molecule and the shortening of an initial fatty acyl-CoA by two carbon atoms [1]. However, the respective reactions, catalysed by acyl-CoA dehydrogenase, enoyl-CoA hydratase, 3-hydroxyacyl-CoA dehydrogenase, and 3-ketoacyl-CoA thiolase, can operate in a biosynthetic direction utilising acetyl-CoA as a key precursor metabolite [2]. Thus, the inverted fatty acid β-oxidation (iBOX) may serve as a versatile biochemical platform for biosynthesis, by directly engineered microbial strains, of a wide array of industrially valuable chemicals, including carboxylic acids, n-alcohols, 2,3-unsaturated carboxylic acids and alcohols, 3-hydroxycarboxylic acids, 1,3-diols, 3-oxocarboxylic acids, and 1,3-ketoalcohols (Figure 1) from cheap and convenient carbon sources such as plant-biomass-derived sugars. The formation of the respective compounds from iBOX intermediates can be ensured in the engineered strains by the action of appropriate terminating enzymes, i.e., thioesterases hydrolysing thioester bonds to give carboxylic acids, and aldehyde/alcohol dehydrogenases reducing CoA-thioesters to alcohols.

In recent years, the successful iBOX-mediated biosynthesis of many products from glucose and glycerol has been demonstrated using industrially relevant *Escherichia coli* and *Saccharomyces cerevisiae* by the overexpression of genes encoding both the pathway and necessary terminating enzymes [3,4]. The operation of the target pathway in the engineered *E. coli* strains has generally been ensured by the overexpression of genes encoding enzymes of aerobic fatty acid β-oxidation, including 3-ketoacyl-CoA thiolases AtoB and FadB, bifunctional 3-hydroxyacyl-CoA dehydrogenase/enoyl-CoA hydratase FadB, and FADH_2_-dependent acyl-CoA dehydrogenase FadE [5,6,7,8,9], although enoyl-ACP reductase FabI, the enzyme of fatty acid biosynthesis with the promiscuous activity towards acyl-CoA substrates, has also been shown to efficiently drive iBOX by catalysing the acyl-CoA dehydrogenase-mediated reaction in an NADH-dependent manner [10,11,12,13]. The production of n-alcohols ranging from n-butanol [5,6] to n-dodecanol [14,15] has been achieved through the overexpression of either alcohol/aldehyde dehydrogenases, such as native *E. coli* AdhE, or the heterologous alcohol-forming acyl-CoA reductases, such as Maqu_2507 from *Marinobacter aquaeolei*. The biosynthesis of 1,3-butanediol by the AdhE-deficient strains has resulted from the action of the collateral cellular oxidoreductases specific to 3-hydroxyfunctionalysed substrates, i.e., MhpF, FucO, and YqhD [8]. The overexpression of medium-chain-specific thioesterase TesA led to the production of C6–C10 carboxylic acids [16], and the overexpression of YdiI supported accumulation of the unsaturated dicarboxylates [17]. Recruiting enoyl-CoA hydratase PhaJ1 and polyhydroxyalkanoic acid synthase PhaC2 from *Pseudomonas aeruginosa* as the terminating enzymes allowed for an efficient polyhydroxyalkanoate formation through iBOX by an engineered *E. coli* strain upon the inactivation of both 3-hydroxyacyl-CoA-specific thioesterase TesB and the nonspecific YciA [18].

Despite the significant progress in iBOX-mediated production of these chemicals, the biosynthesis of only 3-hydroxybutyrate (3-HBA) as the main or side product of β-oxidation reversal has been reported in a limited number of studies in the case of 3-hydroxycarboxylic acids [7,19,20]. Short- and medium-chain-length 3-hydroxycarboxylic acids could be used as precursors for the production of various fine chemicals, including drugs, perfumes, and pheromones [21,22,23,24], and serve as convenient starters for the catalytic production of gasoline-range alkanes [25]. Thus, the aim of the current study was to evaluate and improve the biosynthesis of 3-hydroxycarboxylic acids from glucose through iBOX by directly engineered *E. coli* strains.

We have previously engineered the derivatives of 3-HBA producing *E. coli* strain BOX3.3 Δ4 (MG1655 *lacI^Q^*, ∆*ackA-pta*, ∆*poxB*, ∆*ldhA*, ∆*adhE*, ∆*fadE*, P_L_-SD_φ10_-*atoB*, P*_trc-_*_ideal-4_-SD_φ10_-*fadB*, P_L_-SD_φ10_-*tesB*, ∆*yciA*) [20] for anaerobic synthesis from glucose of C4–C8 carboxylic acids through iBOX by the overexpression of *fadE* and *fabI* genes. In the present work, we report the examination of the microaerobic production of the 3-hydroxycarboxylic acids by the respective strains, BOX3.3.1 and BOX3.3.2, and the improvement in their biosynthetic performance by directed manipulation of the expression of the genes encoding key enzymes responsible for iBOX operation. As a result of this proof-of-concept study, we demonstrate the biosynthesis of 14.5 mM of even-chain-length C4–C8 hydroxycarboxylic acids from glucose through iBOX by the engineered *E. coli* strains with a total yield of 0.34 mol/mol and a C4/C6/C8 ratio averaging approximately 63/28/9. The biosynthetic production of C4+ 3-hydroxycarboxylic acids from a glycolytic carbon source through iBOX was demonstrated for the first time in the current work using directly engineered *E. coli* strains.

## 2. Materials and Methods

### 2.1. Bacterial Strains, Plasmids, and Media

The bacterial strains and plasmids used in this study are listed in Table 1. *E. coli* K-12 strain MG1655 served as the parent for all recombinants engineered in this study.

Chromosomal modifications were performed using the lambda Red recombineering technique [26]. The primers that were used are listed in Table S1. A linear DNA fragment for the inactivation of the *tesA* gene, comprising the excisable antibiotic resistance marker, i.e., chloramphenicol acetyltransferase *cat* gene flanked by the lambda phage *attR* and *attL*, was PCR-amplified using primers P1 and P2, and the pMW118-(λ*attL*-Cm-λ*attR*) plasmid [27] was used as a template. The construction of DNA fragments for the replacement of the regulatory regions located upstream of the *fadB*, *fadE*, *fabI*, and *fadA* genes by the artificial genetic element P_L_-SD_φ10_ was performed in several stages as described previously [8]. A DNA fragment containing the *Bgl*II recognition site, P_L_ promoter, and part of the SD of the T7 phage gene φ10 was obtained by PCR using primers P3 and P4, and the genomic DNA of the lambda phage was used as a template. The DNA fragments comprising the *Bgl*II recognition site, λ*attL*-*cat*-λ*attR* cassette, and 36 nucleotides homologous to DNA regions upstream of the coding regions of the *fadB*, *fadE*, *fabI*, and *fadA* genes were PCR-amplified using the plasmid pMW118-(λ*attL*-Cm-λ*attR*), serving as a template, and primer pairs P5 and P6, P5 and P7, P5 and P8, and P5 and P9. The resulting DNA fragments were treated with *Bgl*II, ligated with T4 DNA ligase, and then PCR-amplified using primer pairs P6 and P10, P7 and P11, P8 and P12, and P9 and P13. Primers P10-P13 contained the regions complementary to the 3`-end of the P_L_-SD_φ10_, and the first 36 nucleotides from the reading frames of the *fadB*, *fadE*, *fabI*, and *fadA* genes.

**Table 1:** Bacterial strains and plasmids used in this study.

Strain/Plasmid	Genotype	Reference
*E. coli* strains		
MG1655	Wild-type *E. coli* K-12 strain VKPM B-6195	VKPM
BOX3.3 Δ4	MG1655 *lacI^Q^*, ∆*ackA-pta*, ∆*poxB*, ∆*ldhA*, ∆*adhE*, ∆*fadE*, P_L_-SD_φ10_-*atoB*, P*_trc-_*_ideal-4_-SD_φ10_-*fadB*, P_L_-SD_φ10_-*tesB*, ∆*yciA*	[20]
BOX3.3.1	MG1655 *lacI^Q^*, ∆*ackA-pta*, ∆*poxB*, ∆*ldhA*, ∆*adhE*, P_L_-SD_φ10_-*atoB*, P*_trc-_*_ideal-4_-SD_φ10_-*fadB*, P_L_-SD_φ10_-*tesB*, ∆*yciA*, P*_trc-_*_ideal-4_-SD_φ10_-*fadE*	[9]
BOX3.3.2	MG1655 *lacI^Q^*, ∆*ackA-pta*, ∆*poxB*, ∆*ldhA*, ∆*adhE*, P_L_-SD_φ10_-*atoB*, P*_trc-_*_ideal-4_-SD*_φ__10_*-*fadB*, ∆*fadE*, P_L_-SD_φ10_-*tesB*, ∆*yciA*, P*_trc-_*_ideal-4_-SD_φ10_-*fabI*	[9]
BOX3.3.1.0	BOX3.3.1 ∆*tesA*	This study
BOX3.3.2.0	BOX3.3.2 ∆*tesA*	This study
BOX3.3.1.1	BOX3.3.1 P_L_-SD_φ10_-*fadB*	This study
BOX3.3.2.1	BOX3.3.2 P_L_-SD_φ10_-*fadB*	This study
BOX3.3.1.2	BOX3.3.1 P_L_-SD_φ10_-*fadB*, P_L_-SD_φ10_-*fadE*	This study
BOX3.3.2.2	BOX3.3.2 P_L_-SD_φ10_-*fadB*, P_L_-SD_φ10_-*fabI*	This study
BOX3.3.1.3	BOX3.3.1 P_L_-SD_φ10_-*fadB*, P_L_-SD_φ10_-*fadE*, P_L_-SD_φ10_-*fadA*	This study
BOX3.3.2.3	BOX3.3.2 P_L_-SD_φ10_-*fadB*, P_L_-SD_φ10_-*fabI*, P_L_-SD_φ10_-*fadA*	This study
Plasmids		
pKD46	pINT-ts, *bla*, P*_araB_*-λ*gam*-*bet-exo*	[26]
pMW118-(λ*attL*-Cm-λ*attR*)	pSC101, *bla*, *cat,* λ*attL*-*cat*-λ*attR* cassette	[27]
pMWts-Int/Xis	pSC101-ts, *bla*, P_R_-λ*xis-int*, c*I*ts857	[28]

The respective DNA fragments were integrated separately into the chromosome of the *E. coli* MG1655 strain harbouring the pKD46 [26] helper plasmid. The deletion of the *tesA* gene in the chromosome was verified by PCR using the locus-specific primers P14 and P15, and the integration of a new regulatory element with the correct structure upstream of the coding regions of the *fadB*, *fadE*, *fabI*, and *fadA* genes was confirmed by sequencing with the primer pairs P16 and P17, P18 and P19, P20 and P21, and P22 and P23.

The individually obtained modifications were combined in the chromosomes of the target strains by P1-mediated transductions, followed by the excision of the antibiotic resistance marker from the chromosomes of the strains using the pMWts-Int/Xis plasmid, as described previously [28].

*E. coli* cells were grown in rich LB, SOB, SOC media, and minimal M9 medium (15.1 g/L Na_2_HPO_4_·12H_2_O, 3 g/L KH_2_PO_4_, 1 g/L NH_4_Cl, 0.5 g/L NaCl, 0.015 g/L CaCl_2_·2H_2_O, 0.25 g/L MgSO_4_·7H_2_O, and 5 mg/L thiamine) [29]. Ampicillin (100 μg/mL) and/or chloramphenicol (30 μg/mL) were added as needed.

### 2.2. Culturing of the Engineered Strains for Microaerobic Production of iBOX-Derived Compounds

The engineered strains were grown overnight in M9 medium containing 2 g/L of glucose at 37 °C. Five millilitres of the overnight culture was diluted ten times with 45 mL of M9 medium containing 10 g/L of glucose and 10 g/L of yeast extract. The cultures were then incubated microaerobically in 750 mL flasks sealed with cotton plugs at 37 °C on a rotary shaker at 250 rpm for 10 h. To induce the gene expression, isopropyl-β-D-thiogalactoside (IPTG) was added to the medium 3 h after inoculation at a final concentration of 1 mM. All experiments were performed in triplicate.

### 2.3. Analytical Techniques

High-performance liquid chromatography was used for the quantification of organic acids and glucose in the culture media. For organic acid measurements, a Waters HPLC system (Waters, Milford, MA, USA) equipped with a Rezex ROA-Organic Acid H+ (8%) ion-exclusion column (300 × 7.8 mm, 8 μm, Phenomenex, Torrance, CA, USA) was used with detection at 210 nm. An aqueous 2.5 mM H_2_SO_4_ solution served as the solvent with a flow rate of 0.5 mL/min. For glucose measurements, the system equipped with a Spherisorb-NH_2_ reversed-phase column (4.6 × 250 mm, 5 μm, Waters, Milford, MA, USA) and a Waters 2414 refractive index detector was used. An acetonitrile/water mixture in a ratio of 75/25 (*v*/*v*) was used as the mobile phase at a flow rate of 1.0 mL/min. Samples were identified by comparing the retention times with those of the corresponding standards.

The concentrations of butyric and hexanoic acids were determined by gas chromatography with a flame ionisation detector as described previously [9]. A GC-2010 Plus gas chromatograph (Shimadzu, Kyoto, Japan) equipped with a Stabilwax-DA capillary column (Restek, Bellefonte, PA, USA) with a length of 30 m, an inner diameter of 0.25 mm, and a film thickness of 0.25 μm was used. Helium served as the carrier gas at a constant flow rate of 1.2 mL/min. The split injection mode was used (1:20 split ratio, injection volume 0.5 μL). The temperature of the injector was set to 150 °C, while the detector was maintained at 250 °C. The column thermostat was programmed as follows: 2 min isothermal hold at 90 °C, ramping to 200 °C at 10 °C/min, followed by a final isothermal hold for 2 min at 200 °C.

The quantification and identification of 3-hydroxybutyric, 3-hydroxyhexanoic, and 3-hydroxyoctanoic acids in culture media were carried out by GC-MS. An Agilent 6890N gas chromatograph (Agilent Technologies, Santa Clara, CA, USA) equipped with a 7683B autosampler and an Agilent 5975 mass selective detector (Agilent Technologies, Santa Clara, CA, USA) was used for the GC-MS analysis. An Agilent DB-5MS fused-silica column (30 m length, 0.25 mm i.d., 0.25 µm film thickness, Agilent J&W, Santa Clara, CA, USA) was used. As a carrier gas, helium was used at a constant flow rate of 1.0 mL/min. The injector temperature was adjusted to 220 °C and the split injection mode (1:10 split ratio, 1.0 µL injection volume) was used. The column oven was programmed to operate as follows: 1 min isothermal hold at 60 °C, ramping to 160 °C at 5 °C/min and then to 250 °C at 20 °C/min, followed by 10 min isothermal hold at 250 °C. Electron ionisation at 70 eV was used as the ionisation method. The mass selective detector was operated in the SIM mode (233 and 191 *m*/*z* for 3-hydroxybutyric acid, 261 and 219 *m*/*z* for 3-hydroxyhexanoic, 289 and 247 *m*/*z* for 3-hydroxyoctanoic acid, 275 *m*/*z* for 3-hydroxyheptanoic acid). The ion source temperature was set to 230 °C and the transfer line temperature to 250 °C. 

Sample preparation comprised the extraction of the analytes from the culture media, the evaporation of the extract, and silylation to the trimethylsilyl derivatives. The target analytes were extracted by liquid–liquid microextraction with ethyl acetate (HPLC grade, Biosolve Chimie, France). Prior to the extraction, 500 μL of the sample freed from biomass by centrifugation was mixed with the aqueous solutions of internal standard (3-hydroxyheptanoic acid) and 50 μL of 2.0 M NaCl (ACS grade, Sigma-Aldrich, Saint Louis, MO, USA), followed by adjustment to pH = 1–2 with 2.0 M HCl (ACS grade, Sigma-Aldrich, Saint Louis, MO, USA). Extraction was performed by two serial additions of 500 µL of ethyl acetate followed by vigorous vortexing (5 min) and subsequent centrifugation. After the extraction step, ethyl acetate extract was mixed with dry sodium sulphate (ACS grade, Sigma-Aldrich, Saint Louis, MO, USA) and centrifuged, and the supernatant was evaporated to minimum volume at 30 °C in an Eppendorf Concentrator Plus (Eppendorf, Hamburg, Germany). The silylation was carried out by incubation of dry residue with bis(trimethylsilyl)trifluoroacetamide containing 1% trimethylchlorosilane (Sigma-Aldrich, Saint Louis, MO, USA at 60 °C for 15 min. 

Agilent MSD ChemStation software (G1701 EA ver. E.02.02 SP2) was used for data acquisition and processing. The analytical system was calibrated in the range of 1–1000 μg/mL for target analytes and a limit of detection of 0.25 μg/mL was achieved. The calibration data were analysed by weighted linear regression and the coefficient of determination R^2^ = 0.995 was obtained.

## 3. Results and Discussion

### 3.1. Evaluation of the Biosynthesis of 3-Hydroxycarboxylic Acids from Glucose through iBOX by Engineered E. coli Strains

The *E. coli* strain BOX3.3 Δ4 [20] (Table 1) has previously been engineered to produce 3-HBA from glucose resulting from the partial one-turn reversal of the fatty acid β-oxidation pathway. The expression of the *atoB* and *fadB* genes, which code for β-oxidation enzymes acetyl-CoA C-acetyltransferase and bifunctional (*S*)-3-hydroxyacyl-CoA dehydrogenase/enoyl-CoA hydratase, was enhanced in this strain to ensure acetyl-CoA to 3-hydroxybutyryl-CoA conversion, while the *fadE* gene encoding acyl-CoA dehydrogenase was deleted to prevent acyl-CoA formation and, therefore, multiple iBOX turnovers. Thioesterase II encoded by the *tesB* gene was overexpressed and served as the terminating enzyme capable of hydrolysing the thioester bond of 3-hydroxyacyl-CoA molecules with the formation of the corresponding 3-hydroxyfunctionalysed carboxylic acids. The mixed-acid fermentation pathways competing with iBOX for key precursor metabolites, pyruvate and acetyl-CoA, were inactivated in the strain by the deletion of the *ackA*, *pta*, *poxB*, *ldhA*, and *adhE* genes, and the intracellular availability of acetyl-CoA for target reactions was further increased due to the deletion of the nonspecific thioesterase YciA gene [7,20]. As a result, the strain accumulated 4 mM and 6 mM 3-HBA during the anaerobic and microaerobic tube-scale fermentation, respectively [20]. Upon the overexpression in BOX3.3 Δ4 of *fadE* and *fabI* genes encoding key *E. coli* enzymes possessing acetyl-CoA dehydrogenase activity, the corresponding derived strains BOX3.3 Δ4 P*_trc-_*_id-4_-*fadE* and BOX3.3 Δ4 P*_trc-_*_id-4_-*fabI*, designated as BOX3.3.1 and BOX3.3.2 (Table 1), synthesised in the absence of aeration and notable amounts of butyric (BA), hexanoic (HA), and octanoic (OA) acids resulting from the multi-turn iBOX operation [9]. Although the production of 3-hydroxycarboxylic acids by these strains has not been previously assessed, their biosynthetic performance, along with the capability of the progenitor strain BOX3.3 Δ4 to synthesise 3-HBA, suggested that at least C4–C8 3-hydroxycarboxylates could also be produced by these recombinants under appropriate conditions. Thus, BOX3.3.1 and BOX3.3.2 were selected in the current study as the core strains for the evaluation and improvement of the biosynthesis of 3-hydroxycarboxylic acids from glucose through iBOX by directly engineered *E. coli*.

**Figure 2:**
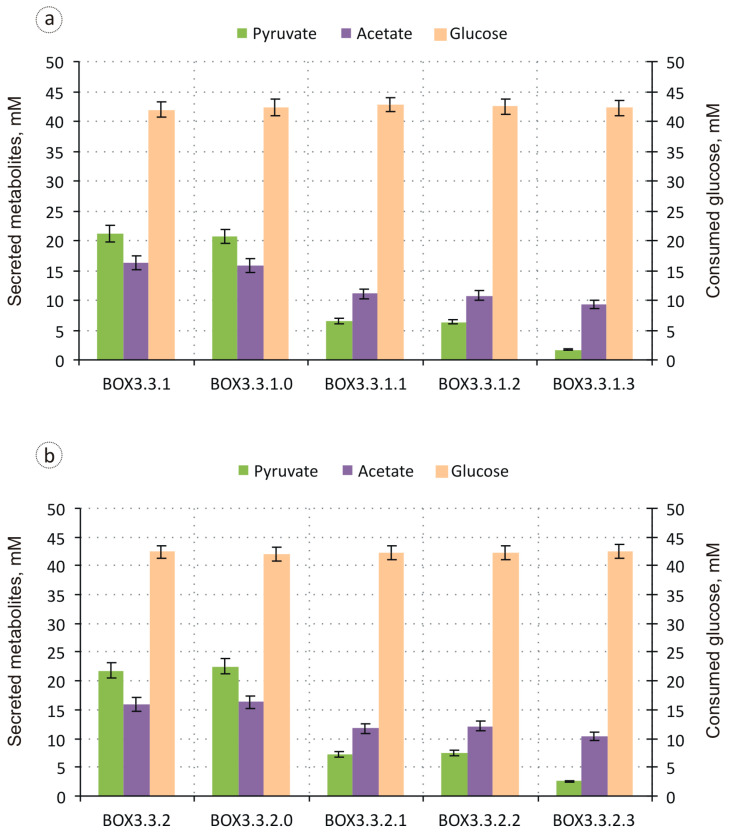
Metabolite production and substrate consumption by the strains BOX3.3.1 (**a**) and BOX3.3.2 (**b**), and the respective engineered derivatives during 10 h of microaerobic glucose utilisation.

Since 3-hydroxyacyl-CoA dehydrogenase and acyl-CoA dehydrogenase both catalyse NADH-consuming reactions, the synthesis of carboxylic acids through iBOX requires two times more NADH than that of the corresponding 3-carboxylic acids (Figure 1). Thus, strict anaerobiosis appears to be more suitable for the production of carboxylic acids, whereas microaerobic cultivation conditions should favour the formation of 3-hydroxycarboxylates. Consequently, the production of metabolites, including iBOX derivatives, by the strains BOX3.3.1 and BOX3.3.2 was initially evaluated during microaerobic glucose utilisation. Both strains secreted almost 45% of consumed glucose as pyruvic and acetic acids (Figure 2) with yields of ~0.5 mol/mol and ~0.38 mol/mol, respectively. 

**Figure 3:**
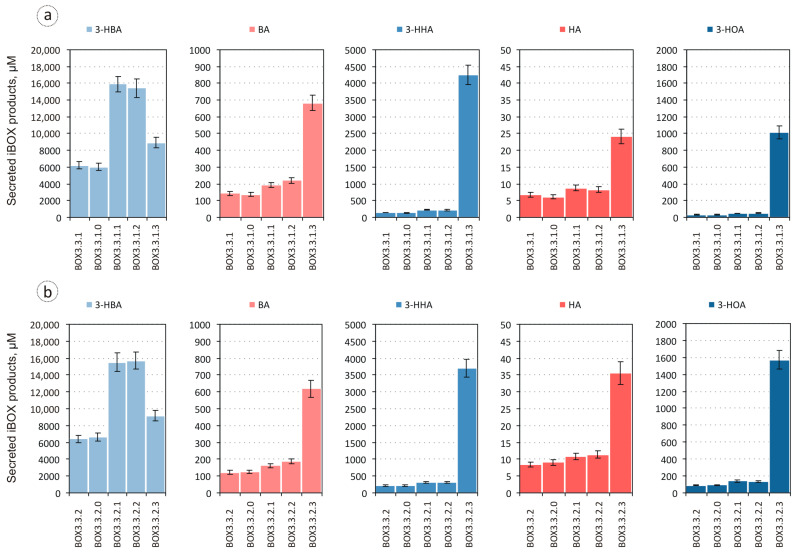
Accumulation of iBOX products by the strains BOX3.3.1 (**a**), BOX3.3.2 (**b**), and the respective engineered derivatives during 10 h of microaerobic glucose utilisation. 3-HBA, 3-hydroxybutyric acid; BA, butyric acid; 3-HHA, 3-hydroxyhexanoic acid; HA, hexanoic acid; 3-HOA, 3-hydroxyoctanoic acid.

The considerable secretion of these acids by the strains was caused by glucose overflow metabolism [30,31] and indicated an insufficient involvement of acetyl-CoA, derived from glycolytically formed pyruvate, to the iBOX reactions. Nevertheless, the strains converted almost 16% glucose to iBOX-derived products, both carboxylic and 3-hydroxycarboxylic acids, with a chain length ranging from four to eight carbon atoms (Figure 3). The most abundant compound among the iBOX-derived products synthesised by the strains was 3-HBA produced with a titre of ~6 mM and a yield of 0.15 mol/mol, followed by 3-hydroxyhexanoic acid (3-HHA) and BA, and 3-hydroxyoctanoic acid (3-HOA) and HA, which were accumulated only in minor amounts. The ratios of the corresponding acids produced by the strains BOX3.3.1 and BOX3.3.2 were slightly different. While the strains synthesised nearly equal amounts of 3-HBA, BOX3.3.2 secreted slightly more 3-HHA and 3-HOA, and less BA compared to BOX3.3.1 (~217 μM, ~90 μM, and ~123 μM vs. ~145 μM, ~33 μM, and ~143 μM, respectively). These results are consistent with previously published data indicating that the overexpression of enoyl-ACP reductase FabI, which possesses NADH-dependent acyl-CoA dehydrogenase activity, was more favourable for multiple iBOX turnovers than the overexpression of ubiquinone-dependent acyl-CoA dehydrogenase FadE [9,10]. The retained secretion by the strains of BA and HA could have resulted from the action of thioesterase I. It has previously been shown that among native *E. coli* thioesterases namely thioesterase I (TesA) preferably hydrolyses the thioester bond of acyl-CoA molecules rather than other CoA derivatives, and the overexpression of the corresponding *tesA* gene in engineered *E. coli* strains supports efficient production of medium- and long-chain carboxylic acids via iBOX [7,16,32]. To verify this assumption, the *tesA* gene was inactivated in the strains BOX3.3.1 and BOX3.3.2. 

The microaerobic production of metabolites from glucose demonstrated by the respective derivative strains BOX3.3.1.0 and BOX3.3.2.0 did not differ significantly from that of the parents (Figure 2 and Figure 3). Thus, thioesterase I, TesA, did not contribute to the formation of carboxylic acids by the engineered strains. *E. coli* possesses at least eight enzymes (FadM, TesA, TesB, PaaI, PaaY, YbgC, YciA, and YdiI) capable of hydrolysing CoA thioesters, but these vary in substrate specificity. While FadM is a long-chain fatty acyl-CoA-specific enzyme [33], PaaI and PaaY preferably recognise phenylacetate degradation intermediates [34], and YdiI exhibits enoyl-CoA thioesterase activity [17], TesB, YciA, and YbgC have relatively broad specificities. Given that the *yciA* gene was inactivated in the BOX3.3 Δ4-derived strains upon *tesB* overexpression, the secretion of BA and HA by the recombinants could be attributed to the promiscuous activity of thioesterase II towards respective acyl-CoA precursors. It should be noted that thioesterase II, TesB, can also utilise acetyl-CoA as the substrate, thus contributing to the observed acetate accumulation by the strains [7,19]. The efficient channelling of the respective thioester towards the iBOX reactions could help to overcome this problem.

### 3.2. Improvement in the Biosynthesis of 3-Hydroxycarboxylic Acids from Glucose through iBOX by Engineered E. coli Strains

The initial design of the model strains BOX3.3.1 and BOX3.3.2. implied an increased efficiency of the iBOX initiation and target product formation over a relatively decreased intensity of the intermediate cycle stages. Accordingly, the native regulatory regions of the *atoB* and *tesB* genes encoding acetyl-CoA C-acetyltransferase and thioesterase II, which are responsible for the primary condensation of acetyl-CoA molecules and the hydrolysis of the thioester bond of CoA-intermediates of the cycle, were replaced in the strains by an artificial genetic element comprising the highly efficient ribosome binding site of gene φ10 from phage T7 and the strong constitutive P_L_ promoter of phage lambda. At the same time, the genes *fadB* and *fadE*/*fabI*, encoding enzymes participating in the formation of 3-hydroxyacyl-CoA, enoyl-CoA, and acyl-CoA, were expressed in strains under the control of the P*_trc_*_-ideal-4_ promoter, which is almost 3 times weaker than the P_L_ promoter [35,36]. Thus, the efficiency of entire β-oxidation reversal in the strains was apparently limited by the deficiency in (*S*)-3-hydroxyacyl-CoA dehydrogenase/enoyl-CoA hydratase and acyl-CoA dehydrogenase activities. Therefore, to improve the functionality of iBOX, the expression of the *fadB* gene was enhanced in the strains BOX3.3.1 and BOX3.3.2 by substituting the P*_trc-_*_ideal-4_ promoter with the P_L_ promoter.

The metabolite profiles formed by the resulting strains BOX3.3.1.1 and BOX3.3.2.1 during the microaerobic utilisation of glucose dramatically changed compared to the core strains (Figure 2 and Figure 3). The production of 3-HBA by the strains rose an average of 2.5 times to ~15.5 mM, and the secretion of 3-HHA and 3-HOA increased 1.5 times to ~224 μM and ~49 μM for BOX3.3.1.1, and to ~315 μM and ~139 μM for BOX3.3.2.1, respectively. Although the production of BA and HA by the strains elevated only 1.1-fold, such improvement in the levels of 3-hydroxycarboxylic acid biosynthesis led to an increase in the total yield of iBOX products to 0.38 mol/mol. Concomitantly, the yields of pyruvate and acetate secreted by the strains dropped nearly 3.3 and 1.5 times. This metabolic shift indicated a drastic increase in the carbon flux through the pyruvate dehydrogenase-catalysed reaction and two initial iBOX reactions leading to 3-hydroxyacyl-CoA formation. Accumulation by the strains of 3-HBA as the main iBOX-derived product instead of longer-chain 3-hydroxycarboxylates suggested, therefore, the limitation in the relative intracellular availability of C4 and C4+ acyl-CoA precursors required for the repeated iBOX turnovers. Since bifunctional enzyme FadB catalyses both 3-hydroxyacyl-CoA and enoyl-CoA formation, this limitation could primarily be caused by the insufficiency of acyl-CoA dehydrogenase activity. To address this bottleneck, the P*_trc-_*_ideal-4_ promoter, which regulates the expression of *fadE* and *fabI* genes, was further substituted in the strains BOX3.3.1.1 and BOX3.3.2.1 with the P_L_ promoter leading to BOX3.3.1.2 and BOX3.3.2.2.

The respective modification had little impact on the biosynthetic performance of the recombinants. The secretion of 3-hydroxycarboxylic acids, HA, and the products of glucose overflow metabolism by the strains BOX3.3.1.2 and BOX3.3.2.2 remained almost unchanged, except for BA, which was accumulated in ~15% higher amounts compared to those of the parent strains (~220 μM and ~186 μM vs. ~191 μM and ~162 μM, respectively). This absence of improvement could be caused by several factors.

First of all, the production of iBOX-derived carboxylic acid (i.e., BA and HA) by the strains could be restricted by the intracellular deficiency in suitable thioesterase I activity, TesA, which should be overexpressed in *E. coli* to ensure efficient formation of the corresponding compounds [16]. On the other hand, it could be due to the inefficient formation of necessary acyl-CoA precursors despite the overexpression in the strains of *fadE* and *fabI* genes. In accordance with the obtained results, the activity of thioesterase II in the cells was sufficient enough to support an increased 3-hydroxycarboxylate formation, while the levels of 3-HBA, 3-HHA, and 3-HOA accumulation by the strains was unaffected by the overexpression of acyl-CoA dehydrogenases. This indicated a persistent limitation in carbon flux through iBOX, eventually resulting in a decreased availability of C4 and C4+ acyl-CoAs, which can be used either for the generation of carboxylic acids or the initiation of the subsequent rounds of the cycle. The efficiency of iBOX operation depends on the availability of reducing power, which drives 3-hydroxyacyl-CoA dehydrogenase and acyl-CoA dehydrogenase-mediated reactions in the biosynthetic direction. Thus, diminished NADH supply under microaerobic conditions could contribute to iBOX retardation in the strains. However, the biosynthesis of ~15 mM of 3-HBA through iBOX in the strains BOX3.3.1.2 and BOX3.3.2.2 consumed an equimolar amount of NADH, suggesting that it might be successfully redistributed to support the formation of longer-chain products. Moreover, ~7 mM of pyruvate secreted by the strains could potentially provide the cells with 7 mM of extra NADH when converted to acetyl-CoA by pyruvate dehydrogenase. Taken together, these considerations pointed to an inefficient re-initiation of iBOX in the engineered strains.

In the strain BOX3.3 Δ4 and its engineered derivatives, acetyl-CoA C-acetyltransferase AtoB served as the main 3-ketoacyl-CoA thiolase catalysing the condensation of acyl-/acetyl-CoA primer and acetyl-CoA extender molecules that initiate both primary and consequent rounds of iBOX. Although this enzyme initiates fatty acid β-oxidation reversal [5,6,7,8,9,10], it preferably recognises C2–C4-priming acyl-CoA units [16,37]. The second *E. coli* 3-ketoacyl-CoA thiolase, FadA, has broader substrate specificity with the highest activity towards medium-chain (C6–C10) substrates [37,38]. In *E. coli*, the genes *fadA* and *fadB* apparently constitute a translationally coupled operon with the transcription proceeding in the *fadB* to *fadA* direction [39]. Thus, upon *fadB* overexpression, 3-ketoacyl-CoA thiolase FadA could also participate in the formation of the respective iBOX intermediates in BOX3.3 Δ4-derived strains.

While (*S*)-3-hydroxyacyl-CoA dehydrogenase/enoyl-CoA hydratase, FadB, is catalytically active in a monomeric form, FadA can only act as a component of heterotetrameric trifunctional enzymatic complex FadB_2_FadA_2_ [40]. Accordingly, when the *fadBA* operon was expressed under the control of the artificial regulatory element P_L_-SD_φ10_ that comprises a robust promoter and a highly efficient ribosome binding site, the equimolar biosynthesis of the corresponding proteins could be compromised, resulting in a deficiency of 3-ketoacyl-CoA thiolase activity relative to 3-hydroxyacyl-CoA dehydrogenase activity. This could be due to the potential uncoupling of transcription–translation and suboptimal translation re-initiation resulting from the alterations in the operon regulation and structure [41,42,43]. Consequently, to maintain the proper balance in the formation of FadB and FadA proteins, the regulatory element P_L_-SD_φ10_ was further introduced into the *fadBA* operon in the strains BOX3.3.1.2 and BOX3.3.2.2 upstream of the *fadA* gene.

The resulting strains BOX3.3.1.3 and BOX3.3.2.3, overexpressing both 3-ketoacyl-CoA thiolases, synthesised iBOX-derived 3-hydroxy- and carboxylic acids as the main products of microaerobic glucose utilisation, converting ~43% of the consumed carbon substrate to the corresponding compounds (Figure 2 and Figure 3). The yield of 3-hydroxycarboxylic acids synthesised by the strains reached ~0.34 mol/mol with a decrease in the contribution of 3-HBA and an increase in the share of 3-HHA and 3-HOA. The profiles of 3-hydroxycarboxylates formed by the strains were slightly different. The strain BOX3.3.2.3 secreted 1.5 times more 3-HOA than BOX3.3.1.3 (~1.5 mM vs. ~1 mM), primarily at the expense of decreased 3-HHA accumulation (Figure 3). This reaffirmed the previous observation that overexpression in the engineered strains of FabI as an acyl-CoA dehydrogenase favours the formation of longer-chain iBOX products. It should be noted that the formation of C6–C8 3-hydroxycarboxylic acids by the strains improved by an order of magnitude, whereas the production of C4–C6 carboxylic acids increased only threefold. The latter indicated that upon the intensification in the strains BOX3.3.1.3 and BOX3.3.2.3 of carbon flux through iBOX, efficiently formed C4–C6 acyl-CoA intermediates of the cycle preferably participated in condensation with acetyl-CoA, thus supporting the initiation of the next rounds of the cycle, rather than being subjected to hydrolysis with the formation of the corresponding carboxylic acids. Concomitantly, the specificity of the terminating thioesterase overexpressed in the strains, as well as microaerobic cultivation conditions, favoured the formation of 3-hydroxyfunctionalised carboxylic acids as the main iBOX-derived products. 

Thus, the engineered strains BOX3.3.1.3 and BOX3.3.2.3 could be considered promising model strains capable of producing 3-hydroxycarboxylic acids from glucose through iBOX, whose biosynthetic performance could further be improved. Interestingly, the total amount of C4–C8 3-hydroxycarboxylates, ~14 mM, synthesised in the current study by these strains, overexpressing only native genes, was even higher than that of the corresponding C8–C10 compounds produced by the *E. coli* strain engineered to produce target substances via the iBOX-resembling artificial biochemical pathway and expressing 3-ketoacyl-CoA thiolase PaaJ from *Pseudomonas putida*, ketoreductase–dehydratase FadJ from *E. coli*, trans-enoyl-CoA reductase Ter from *Treponema denticola*, and thioesterase from *Anaerococcus tetradium* [25]. Overexpression of the native genes generally causes less burden to the host compared to heterologous ones and, thus, allows for more flexible optimisation. Thus, the metabolic flux through iBOX may further be shifted in the BOX3.3.1.3 and BOX3.3.2.3 strains towards the preferable formation of shorter- or longer-chain products by the fine-tuning of *atoB* and *fadA* gene expression, as it has previously been shown that the variations in the levels of 3-ketoacyl-CoA thiolase gene expression in the engineered *E. coli* strains resulted in the formation of iBOX products of different chain lengths [10,16]. On the other hand, the inactivation of all known cellular thioesterases, apart from that chosen to ensure the biosynthesis of the target class of iBOX-derivatives, may markedly improve the specificity of the recombinants towards the formation of the desired products [17].

Finally, manipulating intracellular NADH levels in the strains may help channel more glucose carbon through the iBOX reactions by applying both NADH and acetyl-CoA driving forces to the pathway, thus increasing the yield of 3-hydroxycarboxylic acids and preventing overflow-metabolism-driven accumulation of pyruvate and acetate. Notably, the accumulation of pyruvate by the strains BOX3.3.1.3 and BOX3.3.2.3 dropped dramatically concomitantly with the increase in iBOX-derived product formation, while the secretion of acetate continued at a relatively high level. It is known that acetic acid secreted to the medium during glucose utilisation can be re-assimilated by *E. coli* after the depletion of available carbohydrate carbon sources [44,45]. Therefore, the accumulation of this by-product by the strains indicated NADH availability constraints for the efficient involvement of an excessive acetyl-CoA, originating from either glycolytically formed pyruvate or previously excreted acetate, to the NADH-consuming iBOX reactions. Under aerobic conditions, NADH is primarily re-oxidised in *E. coli* via the respiratory electron transport chain with the oxygen as the terminal electron acceptor. Accordingly, decreasing oxygen supply during the cultivation under controlled microaerobic conditions would favour iBOX operation in the strains BOX3.3.1.3 and BOX3.3.2.3 or its further engineered derivatives, leading to an elevated 3-hydroxycarboxylate accumulation at the expense of acetate secretion, as it has previously been shown in the case of 3-HBA production [19,20].

## 4. Conclusions

We evaluated and successfully improved the microaerobic production of medium-chain-length 3-hydroxycarboxylic acids from glucose through the inverted fatty acid beta-oxidation by the model *E. coli* strains previously metabolically engineered to anaerobically produce non-functionalised carboxylic acids through the respective biochemical pathway. While the total amount of C4–C8 3-hydroxycarboxylates synthesised by the core strains amounted to only 6.5 mM with a 3-HBA share of 97%, their engineered derivatives were capable of accumulating up to 14.5 mM of 3-HBA, 3-HHA, and 3-HOA in the ratio averaging approximately 63/28/9. The best performing recombinants synthesised iBOX-derived acids as the main products of the microaerobic utilisation of glucose, and the total yield of 3-hydroxycarboxilates reached 0.34 mol/mol. Whereas the current work represents a proof-of-concept study, the engineered strains can serve as a suitable platform for the construction of highly efficient producers of the target compounds, thus providing a framework for the further development of bio-based processes for the production of valuable even-chain-length 3-hydroxyfunctionalysed carboxylic acids from renewable raw materials.

## Data Availability

The data presented in this study are available in the article.

## Supplementary Materials

The following supporting information can be downloaded at https://www.mdpi.com/article/10.3390/biom14040449/s1, Table S1: Primers used in this study.
