# Supplementary material for: Biosynthesis of C4–C8 3-Hydroxycarboxylic Acids from Glucose through the Inverted Fatty Acid β-Oxidation by Metabolically Engineered Escherichia coli
Source: Biomolecules. 2024 Apr 7;14(4):449. doi: 10.3390/biom14040449 (PMC11048500; doi:10.3390/biom14040449)
Supplement: Supplementary file 1 [file biomolecules-14-00449-s001.zip › biomolecules-2939863-supplementary.pdf]

Table S1.

Primers used in the study.

Primer	Sequence 5`→3`
P1	5`-ttccgtgccgccgcagcggacacgttattgattctg-cgctcaagttagtataaaaaagctgaac-3`
P2	5`-gcgtcgcggttgggatgaataccgtcatcctgcatc-tgaagcctgcttttttatactaagttgg-3`
P3	5`-tgcgac-agatct-ctcacctaccaaacaatgccc-3`
P4	5`-atgtatatctccttc-acggccaatgcttcgtttc-3`
P5	5`-ctagta-agatct-tgaagcctgcttttttatactaagttgg-3
P6	5`-tacgaccagatcaccttgcggattcaggagactgac-cgctcaagttagtataaaaaagctgaac-3`
P7	5`-gtggtcagacctcctacaagtaaggggcttttcgtt-cgctcaagttagtataaaaaagctgaac-3`
P8	5`-caggcacaacaagcatcaacaataaggattaaagct-cgctcaagttagtataaaaaagctgaac-3`
P9	5`-ccggttggcgacctgaaaacggcttaaggagtcaca-cgctcaagttagtataaaaaagctgaac-3`
P10	5`-ccagtcaaggtacagggtgtcgcctttgtaaagcat-atgtatatctccttcacggccaatg-3`
P11	5`-caggacaaccgtagcgagaatactcaaaatcatcat-atgtatatctccttcacggccaatg-3`
P12	5`-ggttaccagaatgcgcttaccggaaagaaaacccat-atgtatatctccttcacggccaatg-3`
P13	5`-ggtgcgaattgcatcgacaatgacaacctgttccat-atgtatatctccttcacggccaatg-3`
P14	5`-gtgaggatggagagttcatgc-3`
P15	5`-caattccactggaacatccgg-3`
P16	5`-cgtgatcagatcggcatttc-3`
P17	5`-gttcggcaatgccatcttcc-3`
P18	5`-catcacaagtggtcagacctcc-3`
P19	5`-cagactgctgataaataagctcac-3`
P20	5`-gcaactatagctactcacagccag-3`
P21	5`-gtaggtgaatgccagttcagctc-3`
P22	5`-gaaggtctgcgtaataaagcg-3`
P23	5`-cttctgcacgcacgttacg-3`
